# A survey of Korean medicine doctors’ clinical practice patterns for autism spectrum disorder: preliminary research for clinical practice guidelines

**DOI:** 10.1186/s12906-018-2162-4

**Published:** 2018-03-13

**Authors:** Jihong Lee, Sun Haeng Lee, Boram Lee, In Jun Yang, Gyu Tae Chang

**Affiliations:** 1 Department of Clinical Korean Medicine, Graduate School, Kyung Hee University, Seoul, 02453 Republic of Korea; 2 Department of Pediatrics of Korean Medicine, Kyung Hee University Hospital at Gangdong, #892 Dongnam-ro, Gangdong-gu, Seoul, 05278 Republic of Korea; 3 Department of Pediatrics of Korean Medicine, Kyung Hee University Korean Medicine Hospital, Kyung Hee University Medical Center, Seoul, 02447 Republic of Korea; 4 Department of Physiology, College of Korean Medicine Dongguk University, Gyeongju, 780-714 Republic of Korea

**Keywords:** Autism spectrum disorder, Health surveys, Korean traditional medicine, Complementary therapies

## Abstract

**Background:**

The aim of this study was to investigate autism spectrum disorder (ASD) clinical practice patterns of Korean medicine doctors (KMDs) through questionnaire survey.

**Methods:**

Questionnaires on Korean medicine (KM) treatment for ASD were distributed to 255 KMDs on December 5, 2016. The KMDs were psychiatrists, pediatricians, or general practitioners, who treated patients with ASD. The questionnaire covered items on treatment methods, aims of treatment, KM syndrome differentiation, diagnostic tools, and sociodemographic characteristics. Frequency analysis was conducted to describe the participants and their practices.

**Results:**

A total 22.4% KMDs (*n* = 57/255) completed the questionnaires and 54 KMDs (21.2%) matched the inclusion criteria. The KMDs utilized herbal medicine (27.3%), body acupuncture (17.6%), scalp acupuncture (10.7%), moxibustion (6.4%), and Korean medical psychotherapy (5.9%) to treat ASD. The most commonly prescribed herbal medicine was Yukmijihwang-tang. Forty-eight (88.9%) KMDs responded that they used KM syndrome differentiation. ‘Organ system, Qi, Blood, Yin, Yang, Fluid and Humor diagnosis’ was most frequently used for syndrome differentiation. ASD was mainly diagnosed based on the fourth edition of the Diagnostic and Statistical Manual for Mental Disorders (DSM-IV) and DSM-5.

**Conclusions:**

The present study demonstrated the current status of KMDs’ diagnosis and treatment of ASD. In future clinical trials and clinical practice guidelines, these findings will provide meaningful information on the actual practice patterns of KMDs.

**Electronic supplementary material:**

The online version of this article (10.1186/s12906-018-2162-4) contains supplementary material, which is available to authorized users.

## Background

Autism spectrum disorder (ASD) is a lifelong, neurodevelopmental disorder characterized by deficits in social interaction and communication, and restricted and repetitive patterns of behavior, interests, and activities [1]. Approximately 1 in 68 children has been identified with ASD in the United States, with an average prevalence of 1–2% in Asia, Europe, and North America [2]. The prevalence of ASD in a total population sample of South Korean children aged 7–12 years was estimated to be 2.64% [3]. There is no known widely accepted treatment method, but interventions may improve symptoms. Treatment modalities for ASD are categorized as behavioral and educational interventions, psychopharmacologic interventions, and complementary and alternative medicine (CAM) [4].

Korean medicine (KM) has developed similarly to East Asian traditional medicine, such as traditional Chinese medicine and Oriental medicine in Japan [5]. In parallel with the medical system in South Korea, Korean medicine doctors (KMDs) are licensed to independently practice all main curative methods of Korean medical treatments, unlike medical doctors (MDs) who utilize conventional medicine [6, 7]. The main KM therapeutic methods include acupuncture, moxibustion, cupping, herbal medicines, and manual therapies [6]. The KM training system is similar to that of conventional medicine, with 6 years of undergraduate education and a national licensing examination [8]. In 2013, there were 21,355 KMDs in South Korea. The percentage of KMDs among total doctors (including KMDs, MDs, and dental doctors) is 13.5% [9]. The KM specialist training system was introduced in 1999 by the Ministry of Health and Welfare, with reference to the conventional medicine specialist system. After getting a KM license, KMDs choose to train in a designated KM hospital for 4 years (1-year internship and 3-year residence course) and can obtain specialist certification [6]. KMDs with specialist license accounted for 14.3% of all KMDs by 2015 [10]. The types of specialty subjects include internal medicine, acupuncture and moxibustion, rehabilitation medicine, pediatrics, gynecology, ophthalmology, otorhinolaryngology, dermatology, neuropsychiatry, and Sasang constitutional medicine. In 2012, the medical expenses for KM were 4 billion USD, and the proportion of National Health Expenditure was 4.5% [11].

Clinical practice guidelines (CPGs) assist healthcare providers and patients in making treatment decisions [12]. A CPG for ASD has not yet been established in both KM and conventional medicine fields in Korea. KMDs use herbal medicine and acupuncture to treat patients with ASD [13, 14]. However, KM treatment for ASD is not standardized, since KMDs use different KM syndrome differentiations and therapeutic methods. KMDs have difficulty obtaining conclusive evidence for practice, and thus, there is a need to develop evidence-based CPGs for ASD that follow a systematic methodology. The CPGs are required to reflect the current state of KM treatment. The aim of the current study was to investigate ASD clinical practice patterns of KMDs through a questionnaire survey.

## Methods

### Subjects

We conducted a survey targeting KM pediatrics and neuropsychiatry specialists, and general practitioners who stated that they treat patients with ASD. KM pediatrics professors or KM neuropsychiatry professors at KM universities in Korea were also included. We contacted KM pediatrics and KM neuropsychiatrists through the Society of Korean Medicine Pediatrics and Society of Korean Medicine Neuropsychiatry, respectively. In addition, to avoid missing KMDs who treat ASD among other specialists or general practitioners, we performed an additional internet search. To identify KMDs who treat ASD, we searched Google [15], Naver [16], and Daum [17], which are Korean internet search engines, in September 2016. The search phrases, in Korean, were “Autism AND Korean Medicine” and “Autism AND Korean Medicine Clinic”. We visited the websites of the KM clinics to identify pediatric or psychiatric disease clinics, and ascertained whether they provide medical care to patients with ASD. Information about KMDs and KM clinics were collected from the website. Respondents stating that they had no experience in ASD outpatient treatment or for whom we could not obtain the consent form were excluded from the analysis.

### Initial draft

The questionnaire was constructed after a comprehensive literature review and reviewed by KMD researchers. Four participating KMDs had completed 6-year undergraduate courses. One of them (CGT) is a KM pediatrics specialist with more than 20 years of clinical experience and is a professor of KM pediatrics. Two KMDs (LJ and LSH) are KM pediatrics specialists with more than 8 years of clinical experience. The fourth KMD is a resident (LB) majoring in KM pediatrics with more than 2 years of clinical experience. The questionnaire covered items on treatment methods, aims of treatment, KM syndrome differentiation, diagnostic tools, and sociodemographic characteristics. Items regarding KM syndrome differentiation and non-pharmacological treatments were based on the KM neuropsychiatry textbook [18]. One question regarding the division of patient-age group was based on the KM pediatrics textbook [19].

### Second draft

A panel of six extramural experts reviewed and provided advice on the initial draft. The panel was composed of 2 KM pediatric professors at KM universities who were licensed KMDs and four KMDs who mainly treated children or adults with neuropsychiatric disease at KM clinics. One had a Master’s in public health. Four researchers revised the questionnaire based on comments from the panel. The survey questions were finalized after two conferences addressing revisions. An additional file shows the full survey [see Additional file 1].

### Distribution and collection of questionnaires

The questionnaires were distributed and collected directly, by e-mail or registered mail, at the conference site of the Society of Korean Medicine Pediatrics. The e-mails to KM pediatrics and neuropsychiatry specialists were delivered with permission from the Society of Korean Medicine Pediatrics and the Society of Korean Medicine Neuropsychiatry. Registered mail was used for KMDs working at KM clinics for pediatric disease or psychiatric disease; correspondence was accompanied by a cover letter and freepost reply envelope.

The present study was approved by the institutional review board of Kyung Hee University Korean Medicine Hospital at Gangdong in Korea (KHNMCOH 2016–11–008-002). All investigators adhered to the Helsinki Declaration. Respondents signed written informed consent forms to participate, explaining research background, purpose, methods, and personal information protection.

### Statistical analyses

Using descriptive statistics, categorical data were presented by frequencies (percentages, %) to describe the participants and their practices. Items that allowed multiple responses were evaluated using multiple response analysis. Results were analyzed with Microsoft Office Excel version 14.0 (Microsoft Corporation, Redmond, WA, USA).

## Results

A total of 22.4% KMDs (*n* = 57/255) completed the questionnaire (16 face-to-face, 25 by registered mail, and 16 by e-mail). However, two respondents did not return the consent forms, and one was in the residency program and had never treated patients with ASD. Thus, 54 KMDs (21.2%) matched the inclusion criteria.

In the current study, 61.1% of respondents were men, 48.1% were aged 30–39 years, and 44.4% had 10–19 years of experience as a KMD. A total 83.3% were specialists certified by the Korean Ministry of Health and Welfare. Among them, 60% were certified by the Society of Korean Medicine Neuropsychiatry, and 40% were licensed by the Society of Korean Medicine Pediatrics. For most KMDs (83.3%), the proportion of patients with ASD among the total outpatients was less than 5%. The main age group of patients treated by respondents was aged 6–10 years (47.7%) (Table 1).

**Table 1:** Sociodemographic characteristics of surveyed Korea medicine doctors

Factors		N (%)
Age (years)	20–29	0 (0)
	30–39	26 (48.1)
	40–49	23 (42.6)
	50–59	5 (9.3)
	≥60	0 (0)
Sex	Male	33 (61.1)
	Female	21 (38.9)
Clinical experience (years)	≤4	3 (5.6)
	5–9	16 (29.6)
	10–19	24 (44.4)
	≥20	11 (20.4)
Level of healthcare facility of currently affiliated institution	Primary healthcare institution	26 (48.1)
	Secondary healthcare institution	28 (51.9)
Place of work	Capital	21 (38.9)
	Metropolitan city	10 (18.5)
	City	23 (42.6)
	Country	0 (0)
Specialist training	Yes	45 (83.3)
	No	9 (16.7)
Medical specialty	The Society of Korean Medicine Neuropsychiatry	27 (60)
	The Society of Korean Medicine Pediatrics	18 (40)
Rate of autism spectrum disorder patients among total outpatients (%)	< 5	45 (83.3)
	5–9	5 (9.3)
	10–19	2 (3.7)
	20–49	1 (1.9)
	≥50	1 (1.9)
Age of patients with ASD treated by respondents (years) (multiple responses allowed)	< 3	7 (8.1)
	3–5	27 (31.4)
	6–10	41 (47.7)
	11–20	9 (10.5)
	≥21	2 (2.3)

KM syndrome differentiation was generally used (88.9%) in the diagnostic process. The most frequently answered KM syndrome differentiation theory was ‘Organ system, Qi, Blood, Yin, Yang, Fluid, and Humor diagnosis’ (68.2%), followed by ‘Four-constitutional medicine diagnosis’ (18.2%), and ‘Six meridian diagnosis’ (10.6%). Of the titles of syndrome differentiation on ASD, ‘Heart deficiency with timidity (心虛膽怯)’ (12.8%) was most frequently used, followed by ‘Liver qi depression (肝鬱氣結)’ (10%), and ‘Phlegm confounding the orifices of the heart (痰迷心竅)’ (10%). ASD was commonly diagnosed based on the fourth edition of the Diagnostic and Statistical Manual for Mental Disorders (DSM-IV; 26.7%) and DSM-5 (23.8%) (Table 2).

**Table 2:** Korean Medicine syndrome differentiations and diagnostic tools used for autism spectrum disorder

Factors		N (%)
Do you use Korean Medicine syndrome differentiation?	Yes	48 (88.9)
	No	6 (11.1)
Korean Medicine syndrome differentiation theories (multiple responses allowed)	Organ system, Qi, Blood, Yin, Yang, Fluid and Humor diagnosis (臟腑氣血陰陽津液辨證) based on KM textbooks	45 (68.2)
	Four-constitutional medicine diagnosis (四象醫學辨證)’ based on ‘Dongeuisoosebowon (東醫壽世保元)’	12 (18.2)
	Six meridian diagnosis (六經辨證) based on ‘Shang Han Lun (傷寒論)’	7 (10.6)
	Meridian system diagnosis (經絡辨證)	2 (3)
Title of frequently used syndrome differentiation (multiple responses allowed)	Heart deficiency with timidity (心虛膽怯)	23 (12.8)
	Liver qi depression (肝鬱氣結)	18 (10)
	Phlegm confounding the orifices of the heart (痰迷心竅)	18 (10)
	Dual deficiency of heart and spleen (心脾兩虛)	18 (10)
	Qi deficiency (氣虛)	14 (7.8)
	Kidney essence deficiency (腎精虧虛)	14 (7.8)
	Dual deficiency of qi and blood (氣血兩虛)	12 (6.7)
	Liver-kidney yin deficiency (肝腎陰虛)	12 (6.7)
	Kidney qi insufficiency (腎氣不足)	12 (6.7)
	Blood deficiency (血虛)	11 (6.1)
Diagnostic tool (multiple responses allowed)	the Diagnostic and Statistical Manual for Mental Disorders, fourth edition (DSM-IV)	28 (26.7)
	the Diagnostic and Statistical Manual for Mental Disorders, fifth edition (DSM-5)	25 (23.8)
	Not using diagnostic tools but deciding by symptoms	16 (15.2)
	Not using diagnostic tools but referral to other hospital or specialist	15 (14.3)
	Childhood Autism Rating Scale (CARS)	10 (9.52)

The most frequently prescribed KM treatment for ASD was herbal medicine (27.3%), followed by body acupuncture (17.6%), scalp acupuncture (10.7%), and moxibustion (6.4%). The main targets of KM treatment were to alleviate emotional problems (21.7%), improve physical health (18.1%), or improve cognitive ability (16.7%). The most commonly used herbal medicines were Yukmijihwang-tang (12.4%), Ondam-tang (11.2%), and Gwibi-tang (7.7%). Among the single herbs, Acori Graminei Rhizoma (9.5%), Polygalae Radix (8.5%), and Poria (7%) were frequently used. KMDs mainly prescribed compound herbal decoction (55.7%) among the various types of herbal medicine. Herbal medicine for ASD was commonly prescribed to be taken twice a day (61.8%), with a treatment duration of 3–6 months (40.4%), 1–3 months (21.1%), or 6 months to 1 year (21.1%). Regarding acupuncture treatment, GV20 (15.4%), LI4 (11.7%), and Ex-HN1 (Sasinchong, Sishencong) points (8.1%) were the most widely used acupoints. The most commonly applied pharmacopuncture was Hominis Placenta (10.6%) (Table 3). KMDs mainly performed body acupuncture twice (43.8%) or once (37.5%) a week. Scalp acupuncture was also conducted twice (50%) or once (34.6%) a week. The most predominant duration of body acupuncture treatment was 3–6 months (36.6%) or more than 1 year (31.7%). Scalp acupuncture was mainly applied for more than 1 year (34.8%) or 3–6 months (26.1%).

**Table 3:** Frequently prescribed Korean medicine treatments for autism spectrum disorder

Factor		N (%)
Treatment (multiple responses allowed)	Herbal medicine	51 (27.3)
	Body acupuncture	33 (17.6)
	Scalp acupuncture	20 (10.7)
	Moxibustion	12 (6.4)
	Korean medical psychotherapy	11 (5.9)
	Chuna manual therapy	10 (5.4)
	Neurofeedback	7 (3.7)
	Psychotherapy	7 (3.7)
Treatment target (multiple responses allowed)	Emotional problems	48 (21.7)
	Physical condition	40 (18.1)
	Cognitive ability	37 (16.7)
	Behavior problems	34 (15.4)
	Social interaction	33 (14.9)
	Communication ability	27 (12.2)
Herbal medicine (multiple responses allowed)	Yukmijihwang-tang (六味地黃湯)	21 (12.4)
	Ondam-tang (溫膽湯)	19 (11.2)
	Gwibi-tang (歸脾湯)	13 (7.7)
	Eokgan-san (抑肝散)	11 (6.5)
	Sihogayonggolmoryeo-tang (柴胡加龍骨牡蠣湯)	10 (5.9)
	Bojungikgi-tang (補中益氣湯)	8 (4.7)
	Soyo-san (逍遙散)	6 (3.6)
Single herb (multiple responses allowed)	Acori Graminei Rhizoma (石菖蒲)	19 (9.5)
	Polygalae Radix (遠志)	17 (8.5)
	Poria (白茯笭)	14 (7)
	Bupleuri Radix (柴胡)	10 (5)
	Rehmanniae Radix Preparat (熟地黃)	9 (4.5)
	Cyperi Rhizoma (香附子)	8 (4)
	Fossilia Ossis Mastodi (龍骨)	7 (3.5)
	Ostreae Concha (牡蠣)	7 (3.5)
	Angelicae Gigantis Radix (當歸)	7 (3.5)
Acupoint used for acupuncture (multiple responses allowed)	GV20 (百會)	42 (15.4)
	LI4 (合谷)	32 (11.7)
	Ex-HN1 (四神聰, Sasinchong, Sishencong) points	22 (8.1)
	HT7 (神門)	21 (7.7)
	LR3 (太衝)	20 (7.3)
	ST36 (足三里)	18 (6.6)
	PC6 (內關)	16 (5.9)
Pharmacopuncture (multiple responses allowed)	Not using pharmacopuncture	28 (59.6)
	Hominis Placenta (紫河車)	5 (10.6)
	Ginseng (山蔘)	4 (8.5)
	Hwangryeonhaedok (黃連解毒)	4 (8.5)

## Discussion

ASD is a lifetime disease and causes a heavy burden to both families and society. The economic cost for children with ASD in the United States is between 11.5–60.9 billion USD, including healthcare and special education costs, and lost income [20, 21]. No single intervention has been confirmed as being effective in relieving the main symptoms of ASD, which is one reason why families of patients with ASD seek CAM [22, 23]. It was reported that 30–74% of children with ASD used CAM [24–26]. There are published reports on KM treatment for ASD, including herbal medicine and acupuncture, such as case reports [13, 14, 27] and reviews [28, 29]. However, the status of KM or CAM use on children with ASD has not yet been investigated in Korea. The present research was conducted to report the current state of KM treatment for children with ASD.

The rate of ASD among the outpatients that responded was less than 5%, which might be related to the low prevalence of ASD [2, 3]. In addition, KM may be used infrequently to treat ASD in Korea [30–32]. Patients with ASD treated by the respondents were predominantly aged 3–5 or 6–10 years, when multiple responses were allowed. This may have relevant to the fact that most children are not diagnosed with ASD until after 4 years of age [2].

Most KMDs answered that they were using KM syndrome differentiation. Syndrome differentiation is a type of diagnostic method that decides the syndrome based on a group of coexistent symptoms and signs [33]. After syndrome differentiation is decided, the KMDs select the treatment method, including herbal medicine and acupuncture. Syndrome differentiation enables KM treatment to fit individual physical features, and signs and symptoms. When KMDs examine a patient and determine a diagnosis, they significantly consider diseases identification (辨病) as well as syndrome differentiation [34]. In the current study, most KMDs replied that they diagnosed ASD based on DSM-IV and DSM-5. Some KMDs did not use diagnostic tools but decided to conduct the diagnosis based on symptoms. For accurate diagnosis and assessment of treatment, it is recommended that standardized diagnostic tools and appropriate evaluation indexes should be used.

The respondents preferred to use herbal medicine, mostly choosing compound herbal decoction when prescribing herbal medicine. When prescribing compound herbal decoction, KMDs can mix several kinds of herbs in varying amounts, and it can be more effective and helpful for patients with different symptoms and constitutional characteristics. The most frequently prescribed herbal medicine was Yukmijihwang-tang. The memory enhancing and learning function of Yukmijihwang-tang has been demonstrated in laboratory studies [35, 36], with published case studies about herbal medicine containing Yukmijihwang-tang for ASD treatment [13, 14]. Clinical studies on the use of Yukmijihwang-tang in pediatric patients, however need to conducted using rigorous methodology.

Patients with ASD have diverse symptoms, with varying degrees of impairment in social and communication function. KMDs mostly targeted the reduction of emotional problems such as anxiety, irritation, and moodiness. Improving physical condition, associated with food intake, digestion, defecation, and sleep, was also an important therapeutic goal. Gastrointestinal symptoms accounted for a high percentage of the CAM used for children with ASD in the United States [37]. To accurately evaluate the overall therapeutic effect, several assessment indicators for various symptoms will be needed.

Our study has some limitations that need to be noted. One of the most general methods of surveying KMDs is to send e-mails containing a web-based questionnaire to all KMDs through the Associations of Korean Medicine. Nevertheless, this approach is known to have low response rates (3.9–11.1%) [38, 39]. Patients chiefly visit a KM clinic to be treated for musculoskeletal disorders, and the frequency of KM treatment of mental disorders is low in Korea [11]. Thus, we postulated that there was a high probability that general KMDs would not respond to the survey. Therefore, we did not target all KMDs, but sent e-mails to all KM psychiatrists and pediatricians through the Society of Korean Medicine Neuropsychiatry and Society of Korean Medicine Pediatrics, and KMDs who were found by internet searching. Thus, this process may have had a selection bias for KMDs who use e-mails or run a website. Therefore, there may have been some KMDs who treat ASD that were excluded from the analysis. In addition, our study does not show the effect of each of interventions on core symptoms of ASD. Future studies would entail researching the efficacy and safety of specific KM treatments for patients with ASD. Further, the standpoint of families on using KM treatment for ASD has not been examined. Thus, a study on caregiver perceptions about the efficacy, expectation and rationale for using KM treatment is warranted.

The questionnaire employed also has several limitations. There were a lot of items to answer and the length of the questionnaire was long. This may account for missing data. When asked about the type of pharmacopuncture used, 18 KMDs stated the type of pharmacopuncture used, 28 people replied that they did not use pharmacopuncture, and 8 KMDs did not answer this question. We excluded missing data and performed a multiple response analysis. Second, the validity of the questionnaire was partially verified. We searched former survey studies [7, 40, 41] on KM treatment, but did not find validated questionnaires. Accordingly, we derived and modified a considerable number of items from several studies. A panel of experts reviewed the questionnaire, but statisticians or methodologists were not included in the panel. Third, we allowed respondents to provide multiple responses to most items because we intended for the survey to reflect the reality that KMDs judge and change the treatment method according to the circumstances. Another reason for allowing plural responses was that KM treatment was not highly standardized for ASD.

This is the first survey report on clinical practice pattern of KM treatment of ASD. The draft of CPG for KM treatment of ASD was completed in June 2017, and preliminary certification by the Guideline Center for Korean Medicine was obtained in September 2017; it is yet to be published. When establishing a CPG for ASD, the research team reflected on the findings of the current study and made recommendations for each diagnosis and treatment method. Based on the survey results, detailed information was also included that can be used when KMDs treat patients with ASD. In the course of the preparation of the CPG, the research team collected and analyzed practice guidelines and literatures on ASD, and systematically reviewed the efficacy and safety of herbal medicine [42] and acupuncture [43]. In addition, a randomized controlled trial using an herbal medicine, which the present study identified as being widely used, has been scheduled for 2018. The future CPG will be used as an evidence-based guideline in KM institutions, helping KMDs make decisions about ASD and improving the quality and reliability of medical treatment.

## Conclusions

The present study demonstrated the current status of KMDs’ clinical practice patterns for ASD. KMDs commonly used KM syndrome differentiations when diagnosing patients with ASD patients; the ‘Organ system, Qi, Blood, Yin, Yang, Fluid and Humor diagnosis’ was most frequently used among KM syndrome differentiation theories. In addition, herbal medicine and body acupuncture are the most employed KM interventions. Among herbal medicines, Yukmijihwang-tang was the most frequently prescribed. The data of this survey will serve as the basis for CPGs and future clinical trials on ASD. Standardized and reliable CPGs will enable KMDs and patients make rational clinical decisions.

## Additional file

Additional file 1: Survey of Korea Medicine doctors’ clinical patterns for autism spectrum disorder. The final questionnaire answered by KMDs (DOCX 39 kb)

## Abbreviations

ASD: Autism Spectrum Disorder
CAM: Complementary and alternative medicine
CPGs: Clinical practice guidelines
DSM-IV: The fourth edition of the Diagnostic and Statistical Manual for Mental Disorders
KM: Korean medicine
KMD: Korean Medicine doctor
MD: Medical doctor
