# Supplementary material for: A survey of Korean medicine doctors’ clinical practice patterns for autism spectrum disorder: preliminary research for clinical practice guidelines
Source: BMC Complement Altern Med. 2018 Mar 13;18:90. doi: 10.1186/s12906-018-2162-4 (PMC5850908; doi:10.1186/s12906-018-2162-4)
Supplement: Supplementary file 1 — Survey of Korea Medicine doctors’ clinical patterns for autism spectrum disorder. The final questionnaire answered by KMDs (DOCX 39 kb) [file 12906_2018_2162_MOESM1_ESM.docx]

**A survey of Korean medicine doctors’ clinical practice patterns for autism spectrum disorder**

We would appreciate if you could share with us your opinions about Korean medicine treatment for autism spectrum disorder. The contents of this questionnaire will not be used for any purpose other than statistical analysis. Thank you for your sincere reply.

**Ⅰ. Diagnosis** (please √ one box)

**1. Do you use Korean Medicine syndrome differentiation (辨證) when you treat patients with ASD?**

□ Yes **(Go to question 1-1)**

□ No **(Go to question 2)**

**1-1) Which Korean Medicine syndrome differentiation theories do you use** **when you treat patients with ASD? [multiple responses allowed]**

□ Organ system, Qi, Blood, Yin, Yang, Fluid and Humor diagnosis (臟腑氣血陰陽津液辨證)’ based on Korean Medicine textbooks **(Go to question 1-2)**

□ Six meridian diagnosis (六經辨證) based on ‘Shang Han Lun (傷寒論)’ **(Go to question 2)**

□ Meridian system diagnosis (經絡辨證) **(Go to question 2)**

□ Four-constitutional medicine diagnosis (四象醫學辨證) based on ‘Dongeuisoosebowon (東醫壽世保元)’ **(Go to question 2)**

□ Diagnosis based on ‘Hyung Sang medicine (形象醫學)’ **(Go to question 2)**

□ Other (Please specify: ) **(Go to question 2)**

**1-2) Which diagnosis* do you use when you diagnoses based on ‘Organ system, Qi, Blood, Yin, Yang, Fluid and Humor diagnosis (臟腑氣血陰陽津液辨證)’? [multiple responses allowed]**

*Reference: Oriental neuropsychiatry, 3^rd^ edition (The Korean Society of Oriental Neuropsychiatry) and WHO International Standard Terminologies on Traditional Medicine in the Western Pacific Region (World Health Organization).

| □ qi deficiency (氣虛)  □ blood deficiency (血虛)  □ yin deficiency (陰虛)  □ yang deficiency (陽虛)  □ dual deficiency of qi and blood (氣血兩虛)  □ yin deficiency with effulgent fire (陰虛火旺)  □ dual deficiency of qi and yin (氣陰兩虛)  □ deficiency of qi and humor (氣液耗傷)  □ liver-kidney yin deficiency (肝腎陰虛)  □ liver qi depression (肝鬱氣結)  □ depression-heat in the liver meridian  (肝經鬱熱)  □ depressed liver qi transforming into fire  (肝鬱化火)  □ liver hyperactivity and wind stirring  (肝亢風動)  □ heart-liver fire effulgence (心肝火旺) □ heart blood deficiency (心血不足)  □ heart deficiency with timidity (心虛膽怯) □ heart qi deficiency (心氣不足) | □ dual deficiency of heart and spleen  (心脾兩虛)  □ heart-kidney non-interaction (心腎失交)  □ spleen-stomach weakness (脾胃虛弱) □ spleen yang weakness (脾陽虛弱)  □ spleen deficiency with liver effulgence  (脾虛肝旺)  □ spleen-kidney yang deficiency (脾腎陽虛)  □ stomach qi disharmony (胃氣不和) □ lung-spleen qi deficiency (肺脾氣虛)  □ kidney essence deficiency (腎精虧虛) □ kidney qi deficiency (腎氣不足)  □ kidney deficiency and liver hyperactivity  (腎虛肝亢)  □ Phlegm confounding the orifices of the heart (痰迷心竅)  □ phlegm-fire harassing the heart (痰火擾心) □ complicated phlegm and heat (夾熱夾痰)  □ internal harassment of phlegm-heat  (痰熱內搖)  □ stasis-blood obstructing orifice (瘀血阻竅) |
| --- | --- |

**2. Which diagnosis tool do you use when diagnosing a patient with ASD? [multiple responses allowed]**

□ DSM-Ⅳ (the Diagnostic and Statistical Manual for Mental Disorders, fourth edition)

□ DSM-5

□ ABC (Autism Behavior Checklist)

□ CARS (Childhood Autism Rating Scale)

□ GARS (Gilliam Autism Rating Scale)

□ ADI-R (Autism Diagnostic Interview-Revised)

□ ADOS (Autism Diagnostic Observation Schedule)

□ CHAT (Checklist for Autism in Toddlers)

□ Not using diagnostic tool but deciding by symptoms

□ Not using diagnostic tools but referral to other hospital or specialist

□ Other (Please specify: )

**Ⅱ. Treatment**

1. **Check the treatment modality that you apply when treating patients with ASD. [multiple responses allowed]**

| □ Herbal medicine  □ Body acupuncture  □ Scalp acupuncture  □ Tongue acupuncture  □ Auricular acupuncture  □ Electroacupuncture  □ Pharmacopuncture  □ Venesection therapy  □ Moxibustion  □ Cupping  □ Chuna manual therapy (including acupressure and massage therapy)  □ Qigong | □ Korean medical psychotherapy  □ Aromatherapy  □ Physical therapy  □ Language therapy  □ Biofeedback  □ Neurofeedback  □ Sensory integration training  □ Computerized cognitive training  □ Psychotherapy  □ Visual perception training  □ Auditory perception training  □ Other (Please specify)  ( ) |
| --- | --- |

**1-1) What is the treatment target of the treatment modalities checked above? [multiple responses allowed]**

□ To improve social interaction ability (e.g. the ability to form personal relationships, eye contact, the ability to put yourself in another’s shoes, imagination play, etc.)

□ To relieve behavior characteristics (e.g. stereotyped, self-stimulatory behavior, or inappropriate behavior, etc.)

□ To improve verbal and nonverbal communication skills

□ To improve cognitive ability

□ To reduce emotional problems (e.g. anxiety, irritation, moodiness, etc.)

□ To improve physical health (e.g. food intake, digestion, defecation, sleep problems, etc.)

□ Other (Please specify: )

**ⅰ. Herbal Medicine Treatment**

**[Please answer only if you are treating patients with ASD with herbal medicine.]**

**2. Which type of herbal medicine do you prescribe to patients with ASD? [multiple responses allowed]**

□ Compound herbal decoction (湯劑)

□ Distillate of compound herbal decoction (蒸溜湯劑)

□ Powder preparation (散劑)

□ Pill preparation (丸劑)

□ Mixture of soluble granules covered by insurance

□ Soluble granules not covered by insurance

□ Other (Please specify: )

**3. How many times a day do you tell patients with ASD to take herbal medicine?**

□ Four times a day

□ Three times a day

□ Twice a day

□ Once a day

□ PRN (pro re nata)

□ Other (Please specify: )

**4. How long is the average duration of herbal medicine treatment for patients with ASD?**

**[multiple responses allowed]**

□ Less than 15 days

□ 15 to 30 days

□ 1 to 3 months

□ 3 to 6 months

□ 6 months to 1 year

□ More than 1 year

**5. Please fill in the blanks for the most frequently prescribed formula to patients with ASD. [multiple responses allowed]**

| ① |
| --- |
| ② |
| ③ |
| ④ |
| ⑤ |

**6. Please enter the frequently of a prescribed single herb to patients with ASD. [multiple responses allowed]**

| ① | ② |
| --- | --- |
| ③ | ④ |
| ⑤ | ⑥ |
| ⑦ | ⑧ |
| ⑨ | ⑩ |

**ⅱ. Nonpharmacological Treatment**

**[Please answer only if you are treating ASD patients to use nonpharmacological treatment.]**

**7. Please record the number of nonpharmacological treatments per week you perform to patients with ASD. (Check √ box if applicable)**

| **Treatment method** | **Treatment frequency (per week)** | |
| --- | --- | --- |
| □ Body acupuncture |  | **times** |
| □ Scalp acupuncture |  | **times** |
| □ Tongue acupuncture |  | **times** |
| □ Auricular acupuncture |  | **times** |
| □ Electroacupuncture |  | **times** |
| □ Pharmacopuncture |  | **times** |
| □ Venesection therapy |  | **times** |
| □ Cupping |  | **times** |
| □ Chuna manual therapy (including acupressure and massage therapy) |  | **times** |
| □ Qigong |  | **times** |
| □ Korean medical psychotherapy |  | **times** |
| □ Aromatherapy |  | **times** |
| □ Physical therapy |  | **times** |
| □ Language therapy |  | **times** |

**8. How long is the average duration of nonpharmacological treatment for patients with ASD? (Check √ box and mark the average duration if applicable)**

| **Treatment method** | **Average duration**  **1= Less than 1 month**  **2= 1 to 3 months**  **3= 3 to 6 months**  **4= 6 months to 1 year**  **5= More than 1 year** |
| --- | --- |
| □ Body acupuncture | **1 2 3 4 5** |
| □ Scalp acupuncture | **1 2 3 4 5** |
| □ Tongue acupuncture | **1 2 3 4 5** |
| □ Auricular acupuncture | **1 2 3 4 5** |
| □ Electroacupuncture | **1 2 3 4 5** |
| □ Pharmacopuncture | **1 2 3 4 5** |
| □ Venesection therapy | **1 2 3 4 5** |
| □ Cupping | **1 2 3 4 5** |
| □ Chuna manual therapy (including acupressure and massage therapy) | **1 2 3 4 5** |
| □ Qigong | **1 2 3 4 5** |
| □ Korean medical psychotherapy | **1 2 3 4 5** |
| □ Aromatherapy | **1 2 3 4 5** |
| □ Physical therapy | **1 2 3 4 5** |
| □ Language therapy | **1 2 3 4 5** |

**9. Please write down the acupoints that you mainly use for patients with ASD. [multiple responses allowed]**

| ① | ② |
| --- | --- |
| ③ | ④ |
| ⑤ | ⑥ |
| ⑦ | ⑧ |
| ⑨ | ⑩ |

**10. Which type of pharmacopuncture do you use? [multiple responses allowed]**

□ I do not use pharmacopuncture

□ Cervi Pantotrichum Cornu (鹿茸)

□ Hominis Placenta (紫河車)

□ Ginseng (山蔘)

□ Bee Venom (蜂毒)

□ Ganoderma Lucidum (靈芝)

□ Other (Please specify: )

**Ⅲ. Demographic information**

**1. Are you:**

□ Female

□ Male

**2. Your age:**

□ 20-29

□ 30-39

□ 40-49

□ 50-59

□ ≥60

**3. Year of clinical experience:**

□ ≤4

□ 5-9

□ 10-19

□ ≥20

**4. What is your affiliated institution?**

□ Primary healthcare institution (KM clinic/ public health center/hospital under 30 inpatient beds)

□ Secondary healthcare institution (KM hospital with 30 to 500 inpatient beds)

□ Other (Please specify: )

**5. Where is your affiliated institution located?**

□ Seoul Metropolitan City

□ Metropolitan city (□ Busan □ Daegu □ Incheon □ Gwangju □ Daejeon )

□ City (Please specify: )

□ Country (Please specify: )

**6. Specialist training:**

□ Yes (specialist)

□ No (general practitioner)

**6-1) Specialty (if applicable)**

□ Korean medicine Neuropsychiatry

□ Pediatrics of Korean Medicine

□ Korean Medicine Obstetrics and Gynecology

□ Internal Korean Medicine

□ Korean Acupuncture and Moxibustion Medicine

□ Korean Medicine Ophthalmology, Otolaryngology and Dermatology

□ Korean Medicine Rehabilitation

□ Sasang Constitutional Medicine

**7. What percentage of total patients does ASD patients account for?**

□ <5

□ 5-9

□ 10-19

□ 20-49

□ ≥50

**8. Which age group of ASD patients do you usually treat? (years)** **[multiple responses allowed]**

□ <3

□ 3-5

□ 6-10

□ 11-20

□ ≥21
